# α-Glucosidase inhibitors boost gut immunity by inducing IgA responses in Peyer’s patches

**DOI:** 10.3389/fimmu.2023.1277637

**Published:** 2023-11-01

**Authors:** Kisara Hattori-Muroi, Hanako Naganawa-Asaoka, Yuma Kabumoto, Kei Tsukamoto, Yosuke Fujisaki, Yumiko Fujimura, Seiga Komiyama, Yusuke Kinashi, Miki Kato, Shintaro Sato, Daisuke Takahashi, Koji Hase

**Affiliations:** ^1^ Division of Biochemistry, Graduate School of Pharmaceutical Sciences, Keio University, Tokyo, Japan; ^2^ Division of Biochemistry, Department of Pharmaceutical Sciences, Keio University Faculty of Pharmacy, Tokyo, Japan; ^3^ Mucosal Vaccine Project, BIKEN Innovative Vaccine Research Alliance Laboratories, Research Institute for Microbial Diseases, Osaka University, Osaka, Japan; ^4^ Department of Microbiology and Immunology, School of Pharmaceutical Sciences, Wakayama Medical University, Wakayama, Japan; ^5^ The Institute of Fermentation Sciences (IFeS), Faculty of Food and Agricultural Sciences, Fukushima University, Fukushima, Japan; ^6^ International Research and Development Center for Mucosal Vaccines, the Institute of Medical Science, The University of Tokyo (IMSUT), Tokyo, Japan

**Keywords:** follicular helper T cell, immunoglobulin A, peyer’s patch, α-glucosidase inhibitor, voglibose

## Abstract

Peyer’s patches (PPs) are specialized gut-associated lymphoid tissues that initiate follicular helper T (Tfh)-mediated immunoglobulin A (IgA) response to luminal antigens derived from commensal symbionts, pathobionts, and dietary sources. IgA-producing B cells migrate from PPs to the small intestinal lamina propria and secrete IgA across the epithelium, modulating the ecological balance of the commensal microbiota and neutralizing pathogenic microorganisms. α-glucosidase inhibitors (α-GIs) are antidiabetic drugs that inhibit carbohydrate digestion in the small intestinal epithelium, leading to alterations in the commensal microbiota composition and metabolic activity. The commensal microbiota and IgA responses exhibit bidirectional interactions that modulate intestinal homeostasis and immunity. However, the effect of α-GIs on the intestinal IgA response remains unclear. We investigated whether α-GIs affect IgA responses by administering voglibose and acarbose to mice via drinking water. We analyzed Tfh cells, germinal center (GC) B cells, and IgA-producing B cells in PPs by flow cytometry. We also assessed pathogen-specific IgA responses. We discovered that voglibose and acarbose induced Tfh cells, GCB cells, and IgA-producing B cells in the PPs of the proximal small intestine in mice. This effect was attributed to the modification of the microbiota rather than a shortage of monosaccharides. Furthermore, voglibose enhanced secretory IgA (S-IgA) production against attenuated *Salmonella* Typhimurium. Our findings reveal a novel mechanism by which α-GIs augment antigen-specific IgA responses by stimulating Tfh-GCB responses in PPs, and suggest a potential therapeutic application as an adjuvant for augmenting mucosal vaccines.

## 1 Introduction

Follicular helper T (Tfh) cells are a specialized subset of CD4^+^ T cells providing essential assistance to B cells in the secondary lymphoid organs (SLOs). Tfh cells promote the activation and differentiation of B cells into germinal center (GC) B cells, which undergo somatic hypermutation and affinity maturation. GCB cells then differentiate into memory B cells or antibody-secreting plasma cells with sustained support from Tfh cells, thereby generating high-quality humoral immunity (1, 2). The differentiation of Tfh and GCB cells is typically induced by pathogenic infection or vaccination, but their frequency is typically low in most SLOs, except in Peyer’s patches (PPs) of the small intestine (3). PPs are major components of gut-associated lymphoid tissue (GALT). Unlike other SLOs, such as the spleen and lymph nodes, PPs are continuously exposed to numerous antigens derived from food and to commensal microorganisms present in the intestinal lumen. In PPs, antigens are sampled by specialized epithelial cells called microfold (M) cells, triggering the initiation of humoral immune responses involving interactions between Tfh cells and GCB cells (4). In the PPs, GCB cells differentiate into IgA^+^ B cells. Subsequently, they migrate to the mesenteric lymph nodes (MLNs), enter the systemic circulation, and then preferentially return to the small intestinal lamina propria, during which they terminally differentiate into IgA-producing plasma cells (5, 6).

Dimeric IgA produced by plasma cells in the lamina propria is secreted into the lumen by poly-Ig receptors across intestinal epithelial cells. Secretory IgA (S-IgA) is a key immunoglobulin that protects the gut from pathogens by neutralizing toxins and preventing their adherence to epithelial cells, contributing to mucosal homeostasis. S-IgA regulates the composition and function of commensal bacteria (7–9). A large fraction of the S-IgA repertoire is polyreactive, possesses low affinity, and is generated in a T cell-independent manner (10). In contrast, IgA responses specific to pathogenic microbes require Tfh cell support to generate high-affinity S-IgA (11, 12). The distal part of the small intestine harbors a high load of commensal bacteria; thus, a higher number of Tfh and GCB cells is observed in the PPs in this region than in the proximal part (13). Segmented filamentous bacteria (SFB) selectively colonize the distal part of the small intestine by forming intimate contacts with intestinal epithelial cells. Although SFB represent a minor fraction of the gut microbiota, they have a profound influence on host immunity, including inducing Tfh cell differentiation (3, 14). Considering that PPs in the proximal part of the small intestine also contain Tfh cells to a lesser extent, other unknown mechanisms may contribute to the induction of Tfh cells.

α-glucosidases are a family of enzymes that catalyze the cleavage of α-(1→4)-glycosidic bonds at the non-reducing end of oligo- and polysaccharides, releasing monosaccharides that can be absorbed by the small intestinal epithelium (15). This constitutes the final step of carbohydrate digestion in the human gastrointestinal tract. α-glucosidase inhibitors (α-GIs) are a class of antidiabetic drugs that interfere with the enzymatic activity of α-glucosidases, thereby delaying their absorption and lowering the postprandial increase in glucose levels in patients with type 2 diabetes mellitus (16). Since α-GIs inhibit the absorption of carbohydrates from the small intestine, it increases the availability of carbohydrates for fermentation by commensal bacteria, altering the gut microbiota composition (17–19). We hypothesized that these alterations in the commensal microbiota modulate Tfh cell-mediated IgA responses in PPs.

The present study aimed to investigate the effect of α-GIs in IgA responses in PPs. We analyzed Tfh cells, GC B cells, and IgA-producing B cells in the PPs of the proximal and distal small intestine. We also used S. Typhimurium infection as a model to evaluate the effect of α-GIs on pathogen-specific S-IgA production. We provide novel information on how α-GIs augment antigen-specific IgA responses by stimulating Tfh-GCB reactions in PPs. Our findings suggest the potential therapeutic application of α-GIs for augmenting mucosal vaccines as adjuvants

## 2 Materials and methods

### 2.1 Mice

Male and female C57BL/6J mice were purchased from CLEA Japan (total 90 mice, Tokyo, Japan) and housed at the animal facility of Keio University Faculty of Pharmacy (Tokyo, Japan) under controlled conditions (21–22°C, 12-h alternating light–dark cycle). Unless otherwise stated, male mice were used for all the animal experiments. All animal experiments were performed in accordance with the Institutional Guidelines for the Care and Use of Laboratory Animals in Research and were approved by the local ethics committee at Keio University (#A2022-283). The mice were fed an AIN-93G diet (Oriental Yeast, Tokyo, Japan) sterilized with γ-rays. CE-2 chow (CLEA Japan) sterilized with γ-rays was used in some experiments. To evaluate the effect of α-GI, mice were administered drinking water containing 0.0005% or 0.00025% voglibose (Tokyo Chemical Industry, Tokyo, Japan) or 0.125% acarbose (Tokyo Chemical Industry) for 4 weeks. In some experiments, the sucrose contained in the AIN-93G diet was completely replaced with the same amount of D(+)-glucose and D (–)-fructose (nacalai tesque, Kyoto, Japan, **Supplementary Table 1**). In some experiments, mice were administered drinking water containing a cocktail of ampicillin (1 mg/ml, nacalai tesque), neomycin (1 mg/ml, nacalai tesque), vancomycin (0.5 mg/ml, FUJIFILM Wako Pure Chemical Corporation, Osaka, Japan), and metronidazole (0.5 mg/ml, FUJIFILM Wako Pure Chemical Corporation) in the presence or absence of 0.0005% voglibose for 4 weeks. Mice were sacrificed by cervical dislocation under anesthesia with isofluorane inhalation, and small intestine was harvested.

### 2.2 Flow cytometry

PPs from the proximal and distal halves of the small intestine were harvested macroscopically. Single leukocyte suspensions of the PPs were prepared by mechanically disrupting tissues in 2% fetal calf serum (FCS; MP Biomedicals, CA, USA) RPMI1640 media (nacalai tesque) through 100-µm nylon mesh cell strainers (Greiner Bio-One, Kremsmünster, Austria). The leukocytes were pre-incubated with a monoclonal antibody (mAb) against CD16/32 (S17011E; BioLegend, San Diego, CA, USA) in 2% FCS and 0.1% NaN3 in phosphate-buffered saline (PBS) (Nacalai Tesque) before surface antigen staining. Cell staining was performed using mAbs, including Brilliant Violet 786 (BV786)-conjugated anti-CD4 (RM4-5; BD Biosciences, NJ, USA), BV605-conjugated anti-TCRβ-chain (H57-597; BD Biosciences), BV650-conjugated anti-CD25 (PC61; BD Biosciences), BV 510-conjugated anti-CD45 (30-F11; BioLegend, CA, USA), Pacific Blue-conjugated anti-GL-7 (GL7; BioLegend), PerCP-eFluor 710-conjugated anti-PD-1 (J43; Thermo Fisher Scientific, MA, USA), FITC-conjugated anti-IgA (C10-3; BD Biosciences), APC-R700-conjugated anti-CD19 (1D3; BD Biosciences), and APC-conjugated anti-CXCR5 (L138D7; BioLegend) and was followed by dead cell staining with Fixable Viability Stain 780 (FVS780; BD Biosciences). The cells were then fixed, permeabilized, and stained with mAbs, including PE-conjugated anti-Foxp3 (R16-715; BD Biosciences) and PE-CF594-conjugated anti-Bcl-6 (K112-91; BD Biosciences) using a transcription factor buffer set (BD Biosciences). Flow cytometry was performed using a FACSCelesta flow cytometer with DIVA v9.0 (BD Biosciences), and data were analyzed using FlowJo version 10.9 (BD Biosciences).

### 2.3 Fecal IgA enzyme-linked immunosorbent assay (ELISA)

Small intestinal content and feces collected from 7-week-old mice were suspended in PBS containing a Complete proteinase inhibitor cocktail (Roche, Mannheim, Germany) and centrifuged at 12,000 × *g* for 5 min at 4°C. The fecal supernatants were diluted in 2% bovine serum albumin (BSA; globulin free, nacalai tesque)/PBS. Flat-bottomed 96-well MaxiSorp Nunc-Immuno plates (Thermo Fisher Scientific) were coated with a goat anti-mouse IgA antibody (Bethyl Laboratories, TX, USA) for 1 h at room temperature and washed five times with 0.1% Tween 20 in Tris-buffered saline (TBS-T). The plates were then blocked with 2% BSA/PBS at room temperature (20-25°C) for 30 min. The plates were incubated with the diluted samples at room temperature for 1 h. After four washes with TBS-T, horseradish peroxidase-conjugated goat anti-mouse IgA antibody (Bethyl Laboratories) at room temperature for 1 h. The plates were washed five times with TBS-T and incubated with 1-Step Ultra TMB-ELISA Substrate Solution (Thermo Fisher Scientific) for up to 15 min at room temperature. The reaction was stopped by adding 1.2 M sulfuric acid. The absorbance was measured with an Infinite 200 PRO microplate reader (Tecan, Männedorf, Switzerland) at 450 nm.

### 2.4 Immunization and tetanus toxoid (TT)-specific ELISA

Recombinant *Salmonella enterica* subsp. *enterica* serovar Typhimurium (r*Salmonella*)–ToxC (ΔaroA, ΔaroD) was originally developed by Dr. Steve Chatfield (20). Tetanus toxoid (TT) was kindly provided by the BIKEN Foundation (Osaka, Japan). Mice were administered 0.00025% voglibose in drinking water for 2 weeks from 3 weeks of age, and were orally immunized with approximately 5 × 10^7^ colony-forming unit (CFU) of r*Salmonella*-ToxC. Subsequently, 0.00025% voglibose administration was continued and fecal samples were collected one week after immunization. TT-specific IgA in feces was measured using ELISA. Flat-bottomed 96-well MaxiSorp Nunc-Immuno plates were coated overnight with TT (500 ng/well). The plates were blocked with 2% BSA in PBS for 1 h at room temperature and optically diluted fecal extracts were added to the plate wells. TT-specific IgA was measured using the same procedure as that for the total IgA ELISA in section 2.3.

### 2.5 IgA sequencing (IgA-seq)

Mouse feces were homogenized on a 70-μm cell strainer (Greiner Bio-One) using 1% BSA in PBS. After brief centrifugation at 500 × *g* for 5 min at 4°C to remove large debris, the supernatant was centrifuged at 12,000 × *g* for 5 min to pellet the bacteria. The bacterial pellet was resuspended in 1% BSA in PBS containing biotinylated goat anti-mouse IgA (BD Biosciences). After washing with 1% BSA in PBS, the bacteria were stained with streptavidin-BV421 (BioLegend). The bacteria were washed and resuspended in 1% BSA/PBS containing SYTORed (Thermo Fisher Scientific), prior to flow cytometry. Flow cytometric analysis was performed using a FACSAria III flow cytometer with DIVA v9.0 (BD Biosciences), and data were analyzed using FlowJo version 10.9 (BD Biosciences). IgA-binding bacteria were magnetically enriched using a MojoSort system (BioLegend) with streptavidin nanobeads (BioLegend), according to the manufacturer’s instructions.

### 2.6 DNA extraction and 16S rRNA gene sequencing

Genomic DNA was extracted from liver tissue samples using QIAamp Fast DNA Stool Mini Kit (Qiagen, Hilden, Germany) or QIAamp PowerFecal Pro DNA Kit (Qiagen) according to the manufacturer’s instructions. A 16S rRNA genomic library was constructed following the Illumina technical note protocol with some modifications. Briefly, the extracted DNA samples were used as templates for PCR with KAPA HiFi HotStart Ready Mix and primers specific for the 16S rRNA V3–V4 region. The PCR conditions were: initial denaturation at 95°C for 3 min, 35 cycles at 95°C for 30 s, 55°C for 30 s, and 72°C for 30 s, and final elongation at 72°C for 5 min. The amplicons were purified with AMPure XP beads (Beckman Coulter) and dual indices were attached by index PCR with Nextera XT Index kit (Illumina). The libraries were purified with AMPure XP beads and diluted to 4 nM in Tris–HCl buffer. The libraries were pooled and sequenced on MiSeq (Illumina) with 300 bp paired-end reads.

### 2.7 qPCR of SFB

Bacterial genomic DNA was isolated from fecal pellets, and intestinal luminal contents were extracted using a QIAamp Fast DNA Stool Mini Kit (Qiagen). Real-time quantitative PCR (qPCR) was performed to quantify SFB genomic DNA in accordance with a previously published protocol, with some modifications (21). Briefly, 2 µl of 5 ng/µl genomic DNA was added to a mixture of 5 µl SsoAdvanced Universal Probes Supermix, 2 µl sterile water, 1 µl forward primer (5′-TGAGCGGAGATATATGGAGC-3′), reverse primer (5′-CATGCAACTATATAGCTATATGCGG-3′), and probe (5′-/56-FAM/ACT TAGCAG/ZEN/CGAACGGGTGAGTAACA/3IABkFQ/-3′). The qPCR was performed on a CFX Connect real-time PCR analysis system (Bio-Rad, Tokyo, Japan) at 95°C for 3 min with 35 cycles at 95°C for 15 s and 60°C for 20 s. Absolute quantification of bacterial DNA was accomplished using known concentrations of standard DNA. For standard DNA preparation, SFB-specific sequences were amplified using the primers described above. The PCR amplicon was cloned into the pCR-Blunt vector, according to the manufacturer’s instructions.

### 2.8 Microbiome analysis

The FASTQ files were analyzed using the QIIME2 pipeline (QIIME2 version 2020.2). The sequence data were converted to the qza format, demultiplexed and summarized using QIIME2 paired-end-demux. The dada2 plugin of QIIME2 trimmed and denoised the sequences. The feature classifier plugin in QIIME II used a naïve Bayes classifier trained on the SILVA database (version 138) (22) to perform taxonomic assignments. QIIME2 core-metrics-phylogenetic analysis performed diversity analysis. The relative abundance of each taxon was calculated using the taxa collapse QIIME2 plugin.

### 2.9 Data availability

All sequences analyzed in this study were deposited in the DNA Data Bank of Japan (DDBJ) database. The accession number DRA016886 was assigned to the IgA-sequencing data reported in this paper. The accession number DRA016885 was assigned to the 16S rRNA gene sequencing.

### 2.10 Statistical analysis

No statistical methods were used to determine the sample size. Statistical significance between two groups was calculated by Student’s t-test or Welch’s t-test using GraphPad Prism software version 10 (GraphPad). Statistical significance between the control and other groups was calculated using analysis of variance (ANOVA), followed by Dunnett’s multiple comparison test or Dunn’s multiple comparison test. *P* < 0.05 indicated statistical significance.

## 3 Results

### 3.1 α-GIs enhanced Tfh and GCB cell populations, and IgA responses in the PPs of the proximal part of the small intestine

**Figure 1:**
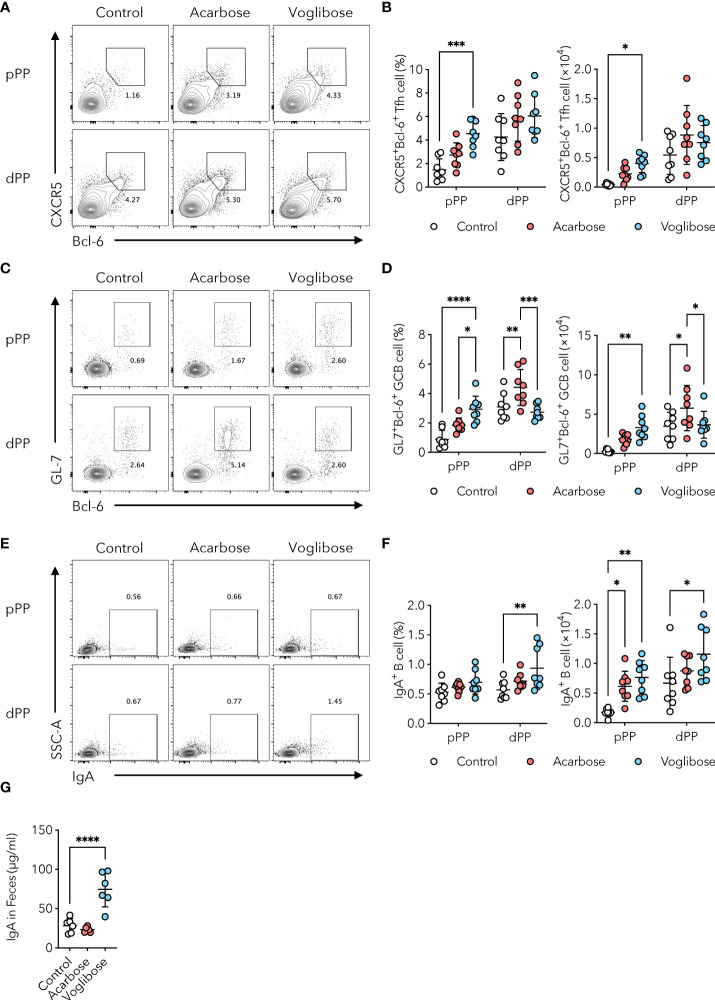
α-GI acarbose and voglibose increase Tfh cells, GCB cells, and IgA production. **(A, B)** Bcl-6^+^CXCR5^+^ Tfh cells in PPs in the proximal and distal part of the small intestine (pPP, dPP, respectively) in the control mice and mice treated with 0.125% acarbose, and 0.0005% voglibose via drinking water. Representative flow cytometry contour plots of Bcl-6 and CXCR5 staining within CD4^+^TCRβ^+^Foxp3^-^CD25^-^ gate **(A)**, and the frequency and total number of Bcl-6^+^CXCR5^+^ Tfh cells **(B)**, *n* = 8, means ± s.d.). **(C, D)** Bcl-6^+^GL-7^+^ GCB cells in pPP and dPP. Representative flow cytometry contour plots of Bcl-6 and GL-7 staining within CD19^+^ gate **(C)**, and the frequency and total number of Bcl-6^+^GL-7^+^ GCB cells **(D)**, *n* = 8, means ± s.d.). **(E, F)** IgA^+^ B cells in pPP and dPP. Representative flow cytometry contour plots of IgA staining within CD19^+^ gate **(E)**, and the frequency and total number IgA^+^ B cells [**(F)**, *n* = 8, means ± s.d.]. **(G)** The concentration of IgA in fecal samples. Mice were administered 0.125% acarbose, and 0.00025% voglibose (*n* = 6, means ± s.d.) via drinking water for 4 weeks starting from 3 weeks of age. Results show one representative experiment of at least two experiments. *P < 0.05, **P < 0.01, ***P < 0.001, ****P<0.0001 [**(B, D, F)**, two-way ANOVA followed by Dunnet’s *post-hoc* test; **(G)**, Student’s *t*-test].

α-GIs modulate the composition and diversity of the commensal microbiota by increasing the availability of carbohydrates for fermentation in the distal part of the intestine (17–19). Since alterations in the composition of commensal microbiota and their metabolites could influence humoral immune responses (23), we initially examined Tfh, GCB, and IgA-producing B cells in PPs, which are the core of gut humoral immune responses, in the distal part of the small intestine. Oral administration of α-GI, namely, acarbose and voglibose, via drinking water had no effect on the number of total CD3ϵ^+^ T cells and CD19^+^ B cells present in the PPs of the distal small intestine (**Supplementary Figures 1A, B**). Similarly, neither acarbose nor voglibose altered the frequency or number of CXCR5^+^Bcl-6^+^ Tfh cells, although an increasing trend was observed (**Figures 1A, B**). In contrast, acarbose increased the frequency and number of GL7^+^Bcl-6^+^ GCB cells, whereas voglibose increased the number of IgA^+^ B cells without affecting the GCB cell population. These data indicate that α-GIs improved GCB and IgA responses in the distal small intestine, but the phenotypes were moderate (**Figures 1C–F**).

Although the number of commensal bacteria was relatively low, we further examined PPs in the proximal part of the small intestine. Voglibose increased the number of CXCR5^+^Bcl-6^+^ Tfh cells by 3.1-fold in frequency and 8.8-fold in number, and acarbose exhibited a similar trend, although it was not statistically significant (**Figures 1A, B**). Voglibose also increased the number of GL7^+^Bcl-6^+^ GCB cells by 3.3-fold in frequency and 11.7-fold in number (**Figure 1C, D**). Neither acarbose nor voglibose changed the frequency of IgA^+^ B cells, but both the α-GIs increased the number of IgA^+^ B cells owing to the expansion of total CD19^+^ B cells (**Figures 1E, F** and **Supplementary Figure 1B**). By aggregating the PPs cell data in the proximal and distal part of the small intestine, we confirmed that the administration of both the α-GIs increased the number of CXCR5^+^Bcl-6^+^ Tfh, GL7^+^Bcl-6^+^ GCB, and IgA^+^ B cells in the PPs (**Supplementary Figures 1C–E**). Consistent with these findings, the voglibose group had higher levels of S-IgA in the proximal small intestinal content and feces than the control group, as shown by a 2.3-fold and 3.9-fold increase, respectively (**Figure 1G** and **Supplementary Figure 1F**). This is in line with the previous findings. On the other hand, acarbose administration did not change the fecal S-IgA levels (**Figure 1G**). Together, these data suggest that voglibose has a substantial ability to enhance IgA responses, particularly in the proximal part of the small intestine, by inducing a Tfh-GCB cell response.

### 3.2 Voglibose altered commensal microbiota composition in the small intestine

**Figure 2:**
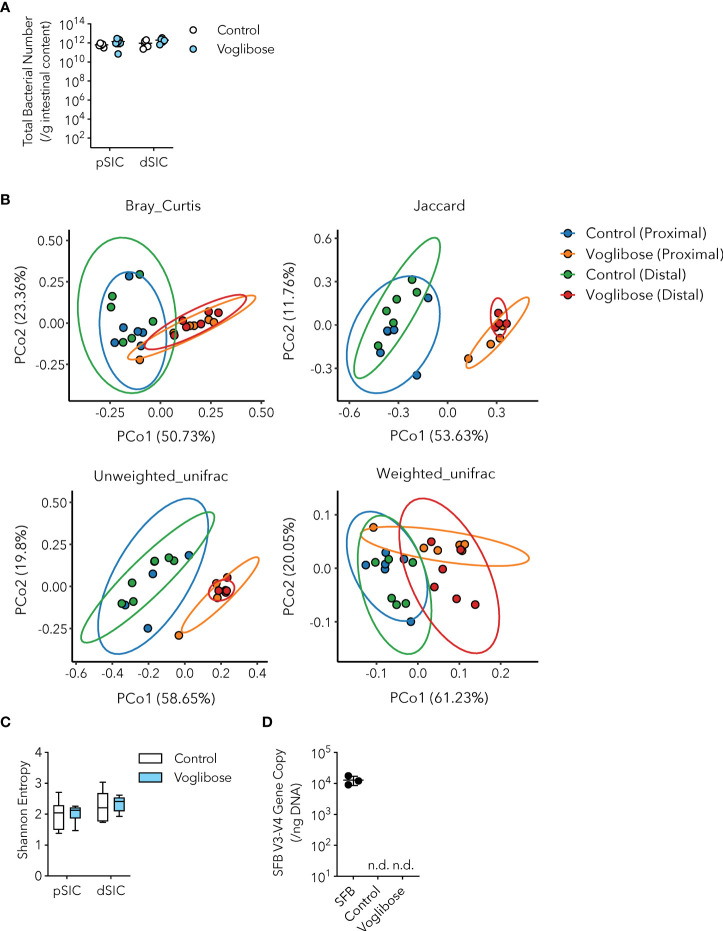
Microbial signature of control and voglibose-treated mice. **(A)** Bacterial number in the proximal and distal small intestinal content of control mice and mice treated with voglibose (pSIC, dSIC, respectively). The bacterial number was assessed by qPCR for 16S rRNA gene sequencing (*n* = 8, means ± s.e.m.). **(B)** Principal coordinate analysis of Bray–Curtis, Jaccard, and unweighted UniFrac, and weighted UniFrac distances among the microbiota in the proximal and distal small intestine of control mice and mice treated with voglibose (*n* = 6). **(C)** Species richness (Shannon entropy) of the microbiota in the proximal and distal small intestine of mice treated with voglibose (*n* = 6, min. to max.) **(D)** Number of SFB in the distal intestinal content of mice mono-colonized with SFB, control mice, and mice treated with voglibose under SPF conditions. The number of SFB was assessed by qPCR for an SFB-specific gene (*n* = 4–6, means ± s.e.m). Mice were administered 0.0025% voglibose via drinking water for 4 weeks starting from 3 weeks of age. Results show one representative experiment of at least two experiments. [**(A, C**, Welch’s *t*-test; **(D)**, one-way ANOVA followed by Dunnet’s *post-hoc* test]. n.d., not detected.

Recent studies have indicated that S-IgA modulates the composition and function of the gut commensal microbiota by aiding or preventing the colonization of certain bacterial species (7). For instance, S-IgA fosters mucosal colonization of human *Bacteroides fragilis* (23). Furthermore, S-IgA alters the expression of mucus-associated functional factors (MAFF) in *Bacteroides thetaiotaomicron*, which facilitates symbiosis with Firmicutes and protects against dextran sulfate sodium (DSS)-induced colitis (24). As voglibose treatment increased the population of IgA-producing B cells in the PPs and fecal S-IgA levels (**Figures 1E–G**), we investigated whether voglibose administration affected the commensal bacterial composition. The number of commensal bacteria is usually very low in the proximal part of the small intestine because of the harsh environment, such as the low pH of gastric juice, high secretion of bile acids, and fast transit time of food (13). α-GIs delay carbohydrate digestion in the host; thus, the population of commensal bacteria increases due to the carbohydrate-rich environment. However, the bacterial density in the intestinal contents of the proximal and distal parts of the small intestine remained unchanged (**Figure 2A**). To investigate the effects of α-GIs on commensal bacterial composition, we performed 16S ribosomal RNA (rRNA) gene amplicon sequencing of the small intestinal contents of mice treated with voglibose. Analysis of β-diversity revealed a clear difference in the composition of microbial communities between the control and voglibose treatment groups, with similar clustering of samples from the proximal and distal part of the small intestine (**Figure 2B**). In contrast, α-diversity exhibited no differences between the RO water and voglibose administration groups in either the proximal or distal part of the small intestine (**Figure 2C**). Moreover, consistent with the change in the β-diversity, voglibose administration resulted in the overrepresentation of the families *Bifidobacteriaceae*, *Erysipelotrichaceae*, and *Akkermansiaceae* in the proximal small intestine (**Supplementary Figures 2A, B**). Voglibose also induced the expansion of *Enterobacteriaceae*, *Erysipelotrichaceae*, and *Akkermansiaceae* in the distal part of the small intestine (**Supplementary Figures 2A, B**). Thus, similar to the effects of other α-GIs, voglibose modulates the composition of commensal bacteria. Additionally, fecal S-IgA level was positively correlated with the relative abundance of *Lactobacillaceae* in the distal part of the small intestine in the mice treated with voglibose (Spearman’s ρ = 0.886, *p* = 0.019; **Supplementary Table 2**), although voglibose administration decreased the relative abundance of *Lactobacillaceae*.

### 3.3 Voglibose-mediated enhancement of Tfh cell responses is dependent on commensal bacteria

**Figure 3:**
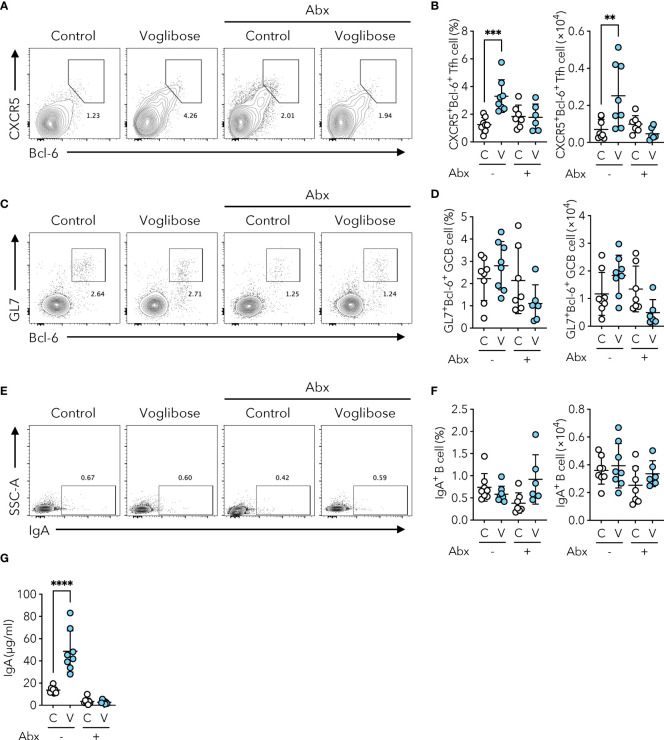
Voglibose increases the population of PP Tfh cells and IgA responses in a microbiota-dependent manner. **(A, B)** Bcl-6^+^CXCR5^+^ Tfh cells in PPs from pPP in control mice (labeled as C) and mice treated with voglibose (labeled as V) with (Abx+) or without antibiotic (Abx-) cocktail. Representative flow cytometry contour plots of Bcl-6 and CXCR5 staining within CD4^+^TCRβ^+^Foxp3^-^CD25^-^gate **(A)**, and the frequency and the total number of Bcl-6^+^CXCR5^+^ Tfh cells **(B)**, *n* = 8, means ± s.d.). **(C, D)** Bcl-6^+^GL-7^+^ GCB cells in pPP. Representative flow cytometry contour plots of Bcl-6 and GL-7 staining within CD19^+^ gate **(C)**, and the frequency and total number of Bcl-6^+^GL-7^+^ GCB cells **(D)**, *n* = 8, means ± s.d.). **(E, F)** IgA^+^ B cells in pPP. Representative flow cytometry contour plots of IgA staining within CD19^+^ gate **(E)**, and the frequency and total number of IgA^+^ B cells **(F)**, *n* = 8, means ± s.d.). **(G)** The concentration of IgA in fecal samples. Mice were administered 0.0005% voglibose via drinking water for 4 weeks starting from 3 weeks of age. An antibiotic cocktail was added to the drinking water. Results show one representative experiment of at least two experiments. **P < 0.01, ***P < 0.001, ****P<0.0001 [**(B, D, F, G)** one-way ANOVA followed by Dunnet’s *post-hoc* test].

Since voglibose markedly enhanced the generation of Tfh, GCB, and IgA-producing B cells in the PPs of the proximal part of the small intestine, we determined whether it was dependent on commensal microbiota. SFB are the only known commensal bacteria that induce Tfh cell differentiation and IgA responses in PPs (3, 25). Thus, we postulated that the α-GI-induced enhancement of Tfh cell differentiation and IgA responses may depend on an increase in SFB colonization. However, SFB levels were barely detectable in the small intestine of mice fed the AIN-93G diet, and voglibose did not alter SFB colonization (**Figure 2D**). These results indicate that the voglibose-induced expansion of Tfh, GCB, and IgA-producing B cells was independent of SFB colonization. To investigate the contribution of commensal bacteria other than SFB to the effects of α-GI, we depleted the bacteria in mice by orally administering a cocktail of antibiotics in drinking water. This treatment did not influence the frequency or number of CXCR5^+^Bcl-6^+^ Tfh cells in the PPs of the proximal small intestine of control mice; however, depleting microbiota canceled the effect of voglibose on CXCR5^+^Bcl-6^+^ Tfh cell induction (**Figures 3A, B**). Similarly, voglibose had no effect on the induction of GL7^+^Bcl-6^+^ GCB or IgA^+^ B cells in the presence of antibiotics (**Figures 3C–F**). Consistent with these results, the concentration of fecal S-IgA was significantly reduced by the antibiotic treatment and remained low after voglibose supplementation (**Figure 3G**). The data presented thus far collectively demonstrate that voglibose increases the number of Tfh, GCB, and IgA-producing B cells in the PPs of the proximal small intestine in male mice in a commensal bacterium-dependent manner. The composition of commensal microbiota exhibits sex-specific differences (26, 27), which are likely due to sex hormones. To investigate whether voglibose induces the expansion of Tfh, GCB, and IgA-producing B cells in female mice, we conducted further experiments. Our results indicate that voglibose significantly increases the number of CXCR5^+^Bcl-6^+^ Tfh cells by 2.4-fold in frequency and 3.9-fold in number in female mice (**Supplementary Figures 3A, B**). Voglibose also increases the number of GL7^+^Bcl-6^+^ GCB cells by 2.3-fold in frequency and 5.9-fold in number in female mice (**Supplementary Figures 3C, D**). Although voglibose has no effect on the frequency of IgA^+^ B cells, it increases the number of IgA^+^ B cells by 4.1-fold in female mice (**Supplementary Figures 3E, F**). Consistent with these results, the voglibose group exhibits higher levels of fecal S-IgA compared to the control group in female mice, as shown by a 3.0-fold increase (**Supplementary Figure 3G**). These data suggest that vogibose enhances Tfh-GCB cell responses and IgA production in a commensal bacteria-dependent fashion regardless of sex differences in commensal bacteria composition. Furthermore, our findings support the notion that voglibose increases PP Tfh cells by modulating the composition of commensal bacteria. Notably, treatment with voglibose had no effect on *in vitro* Tfh cell differentiation (**Supplementary Figure 4**).

### 3.4 Replacement of sucrose with glucose had no effect on voglibose-induced IgA responses

**Figure 4:**
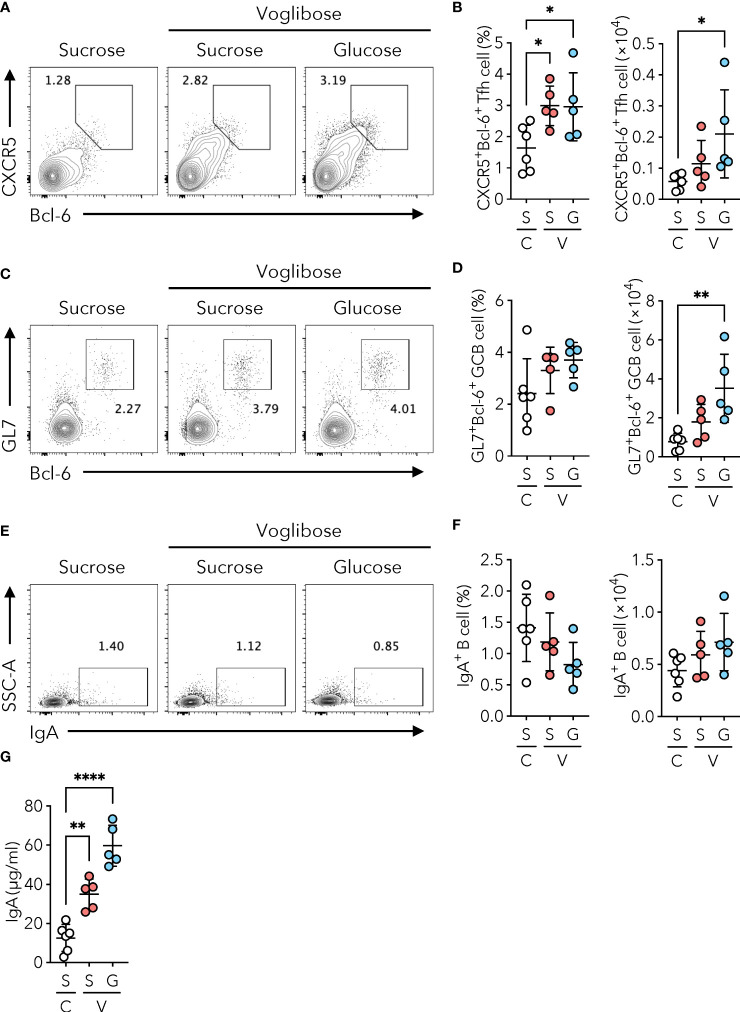
Voglibose enhances PP Tfh cell population and IgA responses independent of α-GI activity. Mice were fed with a sucrose-containing basal AIN-93G diet (labeled as S) or D(+)-glucose-containing AIN-93G (sucrose-free, labeled as G). Complete diet compositions are available in **Supplementary Table 1**. **(A, B)** Bcl-6^+^CXCR5^+^ Tfh cells in pPP of the control mice (labeled as **(C)** and mice treated with 0.0005% voglibose (labeled as V). Representative flow cytometry contour plots of Bcl-6 and CXCR5 staining within CD4^+^TCRβ^+^Foxp3^-^CD25^-^ gate **(A)**, and the frequency and total number of Bcl-6^+^CXCR5^+^ Tfh cells **(B)**, *n* = 8, means ± s.d.). **(C, D)** Bcl-6^+^GL-7^+^ GCB cells in pPP. Representative flow cytometry contour plots of Bcl-6 and GL-7 staining within CD19^+^ gate **(C)**, and the frequency and total number of Bcl-6^+^GL-7^+^ GCB cells **(D)**, *n* = 8, means ± s.d.). **(E, F)** IgA^+^ B cells in pPP. Representative flow cytometry contour plots of IgA staining within CD19^+^ gate **(E)**, and the frequency and total number of IgA^+^ B cells **(F)**, *n* = 8, means ± s.d.). **(G)** The concentration of IgA in fecal samples. Mice were administered 0.0005% voglibose via drinking water for 4 weeks starting from 3 weeks of age. Results show one representative experiment of at least two experiments. *P < 0.05, **P < 0.01, ****P<0.0001 [**(B, D, F, G)** one-way ANOVA followed by Dunnet’s *post-hoc* test].

α-glucosidases are brush border enzymes in intestinal epithelial cells that catalyze the hydrolysis of oligo-, tri-, and disaccharides into glucose and other monosaccharides in the small intestine (15). While some α-GIs such as acarbose target both α-glucosidase and α-amylase, voglibose selectively inhibits maltase, sucrase, and isomaltase, which break down oligo-, tri-, and disaccharides into glucose and fructose (16). We, therefore, postulated that the reduction in the absorption of monosaccharides by the small intestinal epithelium may drive the generation of Tfh, GCB, and IgA-producing B cells in the PPs of the proximal part of the small intestine. To test this hypothesis, we examined whether voglibose-mediated induction of Tfh cells was canceled by additional glucose administration. Because the AIN-93G diet contains sucrose as a source of carbohydrates, we prepared a sucrose-free AIN-93G (basal) diet and a basal diet supplemented with sucrose or α-D-glucose (**Supplementary Table 1**). We confirmed that voglibose administration increased the frequency and number of CXCR5^+^Bcl-6^+^ Tfh cells in the PPs of the proximal part of the small intestine of mice fed sucrose-supplemented basal AIN-93G diet. Notably, voglibose administration also increased the population of PP CXCR5^+^Bcl-6^+^ Tfh cells in mice fed the D(+)-glucose-supplemented AIN-93G diet to a level comparable to that in mice fed the sucrose-supplemented AIN-93G diet (**Figures 4A, B**). Similarly, glucose supplementation increased the number and frequency of GL7^+^Bcl-6^+^ GCB cells (**Figures 4C, D**). Although there was no significant change in the population of IgA^+^ B cells in the PPs of the proximal part of the small intestine (**Figures 4E, F**), the concentration of fecal S-IgA was significantly higher in the voglibose group than in the control group, regardless of the sugar source (**Figure 4G**). By contrast, treatment with D(+)-glucose and D (–)-fructose in the absence of voglibose had no significant effect on Tfh, GCB, and IgA cells, and fecal IgA levels in the proximal small intestinal content (**Supplementary Figures 5A–G**). Collectively, these data indicate that voglibose augmented Tfh-GCB cell and IgA responses independent of α-GI activity.

### 3.5 Voglibose boosted *Salmonella*-specific IgA production

**Figure 5:**
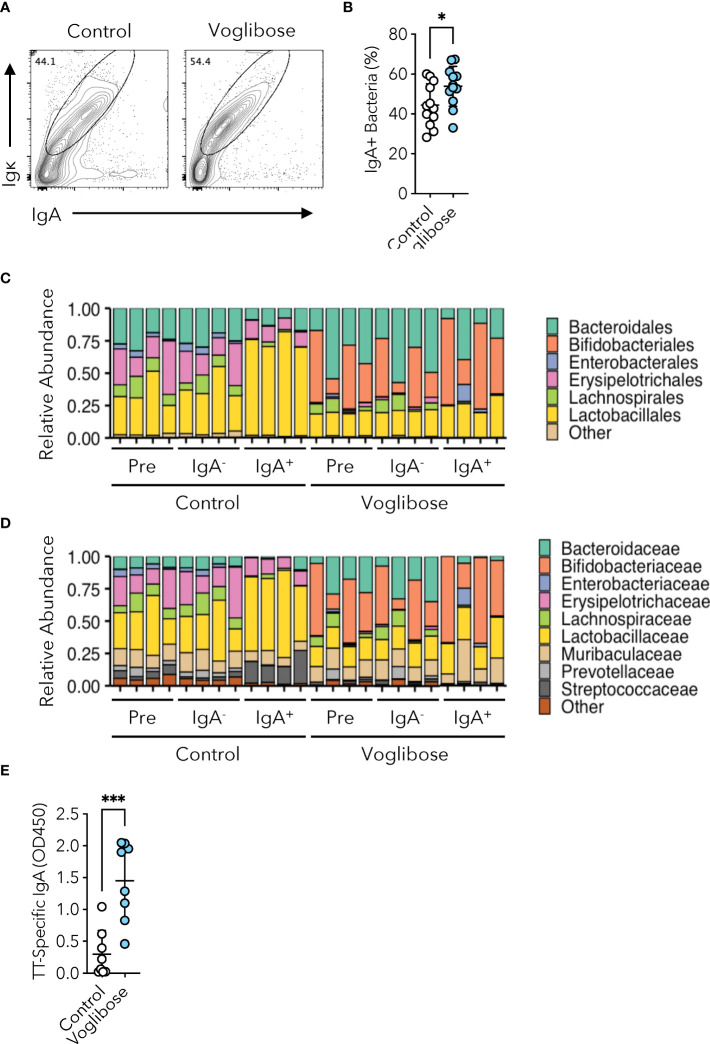
Voglibose enhances commensal and pathogen-reactive IgA responses. **(A, B)** IgA-binding bacteria in feces in the control mice and mice treated with 0.00025% voglibose. Representative flow cytometry contour plots of IgA and Igκ within SYTORed^+^ gate **(A)**, and the frequency of IgA-binding bacteria **(B)**, *n* = 11, 12, means ± s.d.). **(C, D)** The composition of fecal microbiota at the order **(C)** and family **(D)** levels between the control mice and mice treated with 0.00025% voglibose. Pre-sorted (pre), sort purified IgA-binding (IgA^+^), and sort purified IgA non-binding (IgA-) bacterial taxa were shown. **(E)** The concentration of ToxC-specific IgA in fecal samples. Mice were administered 0.00025% voglibose via drinking water for 2 weeks initiating from 3 weeks of age, followed by infection with Salmonella-ToxC (ΔaroA, ΔaroD). Subsequently, administration of 0.00025% voglibose was continued, and fecal samples were collected two weeks after infection. *P < 0.05, ***P < 0.001 [**(B, E)** Welch’s *t*-test].

Given that voglibose administration altered the bacterial composition of the small intestine, we investigated whether S-IgA played a role in these dynamics. Therefore, we aimed to identify the bacterial populations coated with S-IgA using IgA-seq analysis (7, 24). Flow cytometric analysis of fecal IgA revealed that mice administered voglibose had higher levels of microorganism-bound IgA than those in untreated mice (**Figures 5A, B**). IgA-seq analysis revealed that voglibose administration increased S-IgA levels specific to the family *Bifidobacteriaceae*, which was overrepresented in the small intestine of voglibose-administered mice (**Figures 5C, D** and **Supplementary Figures 2A, B**). In the control group, S-IgA-coated bacteria were enriched in the families *Lactobacillaceae* and *Streptococcaceae* (**Figures 5C, D**). Because the majority of S-IgA reacting to commensal bacteria is generated in a T cell-independent manner (10), only a minority of commensal bacteria are responsible for eliciting T cell-dependent S-IgA responses. Tfh cell-mediated GC reactions in the PPs are essential for this process. To investigate whether voglibose administration promotes an antigen-specific IgA response we employed an oral infection model using recombinant *Salmonella* Typhimurium expressing ToxC of the TT (r*Salmonella*-ToxC). r*Salmonella*-ToxC is internalized into PPs by M cells and induces a ToxC-specific humoral immune response (28). We observed that the level of TT-specific fecal IgA increased 7.3-fold in voglibose-treated mice compared with control mice (**Figure 5E**). Flow cytometric analysis demonstrated that voglibose administration increased the frequencies of CXCR5^+^Bcl-6^+^ Tfh and GL7^+^Bcl-6^+^ GCB cells in the PPs of the proximal small intestine after r *Salmonella* -ToxC infection, whereas the numbers of those Tfh and GCB cells were not significantly changed, despite increasing trends (**Supplementary Figures 6A–D**). However, there is no significant change in the frequency and number of IgA^+^ B cells in the PPs of the proximal small intestine between voglibose group and the control group (**Supplementary Figures 6E, F**). Previous studies have demonstrated that commensal bacteria-reactive S-IgA could bind even unmet pathogens including *Salmonella* Typhimurium (29, 30). We therefore hypothesized that voglibose-mediated enhancement of IgA production might prevent the establishment of wild-type (WT) *Salmonella* Typhimurium infection. To this end, we pre-administered voglibose in drinking water for 2 weeks to mice and then orally infected them with a lethal dose of *Salmonella* Typhimurium χ3181. Monitoring of survival demonstrated that voglibose pre-administration had no impact on the survival rate (**Supplementary Figure 7**).

## 4 Discussion

α-GIs increase the availability of carbohydrates for fermentation by commensal gut bacteria and alter their composition. Fecal microbiome analyses have revealed that α-GIs acarbose and miglitol changed the composition of commensal microbiota; however, only a few common changes were observed, probably due to the variation in experimental conditions and methods and differences in basal commensal microbiota composition among the facilities (17–19). In the current study, we observed that an α-GI voglibose altered the composition of the commensal microbiota throughout the small intestine; however, the total bacterial number was not affected (**Figures 2B, C** and **Supplementary Figures 2A–D**). Voglibose administration increased the proportion of the families *Bifidobacteriaceae*, *Erysipelotrichaceae*, and *Akkermansiaceae* in the proximal part of the intestine and that of the families *Enterobacteriaceae*, *Erysipelotrichaceae*, and *Akkermansiaceae* in the distal part of the small intestine, while decreasing the relative abundance of *Lactobacillaceae* in our animal facility (**Supplementary Figures 2A–D**). Characterization of IgA-coated bacterial taxa revealed that voglibose administration increased the relative amount of *Bifidobacteriaceae*-reacting IgA and decreased that of *Lactobacillaceae*-reacting IgA (**Figures 5C, D**). These data suggest that voglibose-induced IgA may selectively promote the colonization of *Bifidobacteriaceae* and/or suppress the colonization of *Lactobacillaceae*, which may contribute to the modulation of the gut microbiota composition and function (7).

It has been widely documented that commensal microbiota shape the host immune system (31). The gut immune system responds to changes in the bacterial community and pathogen infections by adjusting or eliminating them. Tfh–GCB cell axis-mediated IgA production in small intestinal PPs plays an important role in this response. For instance, colonization by SFB drastically enhances Tfh cell differentiation and IgA response in PPs (3, 25). Voglibose administration increased Tfh, GCB, and IgA-producing B cells in the PP, especially in the proximal part, regardless of SFB (**Figures 1A–F**, **2D**), suggesting the involvement of unidentified non-SFB commensal bacteria in PPs Tfh–GCB cell axis-mediated IgA production. Moreover, voglibose administration significantly increased the concentration of IgA in the feces (**Figure 1G**). Commensal bacteria are engulfed by dendritic cells (DCs) in the intestinal lamina propria and are transported to the MLNs, where commensal bacteria-specific IgA responses occur (32). Moreover, even mice lacking PPs induce prime immunized antigen-specific IgA responses, suggesting that MLNs are the major lymphoid tissues involved in the IgA response (33, 34). Thus, it is possible that voglibose enhances IgA responses in the MLNs, which increases fecal IgA levels. However, further studies are required to identify the commensal bacterial taxa involved in the enhancement of Tfh-GCB-mediated IgA responses. Because voglibose administration increased the abundance of the families *Bifidobacteriaceae*, *Erysipelotrichaceae*, and *Akkermansiaceae*, it is possible that commensal bacteria belonging to these families could be the candidates. Additionally, despite voglibose treatment reducing the relative abundance of *Lactobacillaceae*, a positive correlation was observed between the relative abundance of *Lactobacillaceae* in the distal part of the small intestine and fecal S-IgA level in mice treated with voglibose (**Supplementary Table 2**). These findings indicate that Lactobacillaceae may contribute to the enhancement of germinal center responses and subsequent IgA production. However, it is possible that another factor is necessary for this enhancement, such as the co-existence of another bacterial clade, which might be absent in mice that have not been treated with voglibose.

Another α-GI, acarbose, also increased Tfh, GCB, and IgA-producing B cells in PPs while failing to enhance fecal IgA levels (**Figures 1A–G**). Acarbose and voglibose have different selectivity for mammalian maltase, sucrase, and isomaltase. In addition to mammalian α-glucosidases, a previous report demonstrated that voglibose exhibits a strong inhibitory effect on an α-glucosidase from the human gut bacterium *Blaubia obeum* (Ro-αG1), while acarbose barely exhibits it. These two differences could affect commensal bacterial composition, resulting in distinct impacts on PP germinal center responses and IgA production. Moreover, acarbose augments colonic short-chain fatty acid (SCFA) production by increasing starch availability and expanding starch-fermenting microbes (17, 19, 35). We have demonstrated that the SCFA butyrate augments T cell-independent IgA responses in the colon by facilitating the production of intestinal CD103^+^CD11b^+^ DCs (36). Moreover, another SCFA acetate supports T cell-dependent IgA production in the colon by serving as an energy source for CD4^+^ T cells (37). Therefore, it is possible that the increase in IgA level in fecal samples after voglibose administration could be attributed to an increase in SCFA concentration in the colon lumen. Nevertheless, the pathogen-specific IgA response requires PPs, as mice lacking PPs failed to elicit TT-specific IgA responses in the r*Salmonella*-ToxC infection model (38). Our results demonstrated that voglibose administration increased the frequencies of Tfh and GCB cells in the PPs of the proximal small intestine after rSalmonella-ToxC infection (**Supplementary Figures 6A, C**). However, the numbers of Tfh, and GCB cells, as well as the frequencies and numbers of IgA-producing B cells were not affected by vogibose administration (**Supplementary Figures 6A, C**), albeit the increasing tendencies. Nevertheless, considering that voglibose administration significantly increased TT-specific fecal IgA production after r*Salmonella*-ToxC infection (**Figure 5E**), voglibose-induced Tfh–GCB and IgA-producing B cells may be responsible for the pathogen-specific IgA responses. Alternatively, voglibose may create a more favorable niche for r*Salmonella*-ToxC colonization by changing the microbial composition, which contributes to stronger germinal center responses and the subsequent TT-specific IgA generation. Pre-administration of voglibose did not rescue WT *Salmonella* Typhimurium infection-mediated mortality, suggesting voglibose-mediated enhancement of commensal bacteria reactive S-IgA is unable to provide protection against lethal bacterial infections. Nonetheless, there might be a possibility that pre-treatment of the combination of r*Salmonella*-ToxC and voglibose may augment the vaccine effect and reduce the mortality subsequent to WT *Salmonella* Typhimurium infection.

Replacement of sucrose by α-D-glucose did not cancel the enhancement of Tfh and GCB cells by voglibose administration (**Figures 4A–D**). Moreover, the replacement of sucrose by α-D-glucose had no effect on voglibose-induced IgA production in the feces (**Figure 4G**). Our data suggest that voglibose-induced Tfh-GCB responses and the resultant IgA production are independent of α-GI activity of the inhibiting of monosaccharide generation from the small intestinal epithelium. However, further studies are required to clarify the mechanisms by which voglibose enhances Tfh-GCB-mediated IgA responses. Because voglibose is poorly absorbed upon oral administration (39), it is unlikely that it directly affects the function and differentiation of immune cells, including Tfh and GCB cells. Instead, we believe that voglibose-mediated commensal changes are involved in enhancing the Tfh-GCB-mediated IgA responses. However, the mechanism by which voglibose changes the composition of commensal bacteria remains unclear.

In conclusion, the study demonstrated for the first time that α-GI voglibose boosted antigen-specific IgA production in the small intestinal PPs, possibly through enhancing the generation of Tfh–GCB cells. Although the exact mechanisms underlying Tfh-GCB-mediated IgA response enhancement mediated by voglibose administration remain largely unknown, the study provides valuable insights into a new aspect of α-GI as a potential therapeutic agent and adjuvant for augmenting mucosal vaccines. The identification of commensal bacteria involving the generation of Tfh–GCB will help in developing those therapeutic agents and vaccine adjuvants.

## Data availability statement

The data presented in the study are deposited in the DNA Data Bank of Japan (DDBJ) repository, accession number DRA016886 was assigned to the IgA-sequencing data, and accession number DRA016885 was assigned to the 16S rRNA gene sequencing data.

## Ethics statement

The animal study was approved by Keio University Animal Experiment Committee. The study was conducted in accordance with the local legislation and institutional requirements.

## Author contributions

KH-M: Conceptualization, Data curation, Investigation, Methodology, Visualization, Writing – review & editing. HN-A: Conceptualization, Data curation, Investigation, Methodology, Writing – review & editing. YKa: Investigation, Writing – review & editing, Formal Analysis. KT: Investigation, Writing – review & editing. YoF: Investigation, Writing – review & editing. YuF: Investigation, Writing – review & editing. SK: Investigation, Writing – review & editing. YKi: Investigation, Writing – review & editing. SS: Writing – review & editing, Resources. DT: Resources, Writing – review & editing, Conceptualization, Data curation, Formal Analysis, Funding acquisition, Investigation, Methodology, Project administration, Supervision, Validation, Visualization, Writing – original draft. KH: Funding acquisition, Project administration, Supervision, Writing – review & editing. MK: Investigation, Writing – review & editing.
