# Supplementary material for: α-Glucosidase inhibitors boost gut immunity by inducing IgA responses in Peyer’s patches
Source: Front Immunol. 2023 Nov 1;14:1277637. doi: 10.3389/fimmu.2023.1277637 (PMC10646501; doi:10.3389/fimmu.2023.1277637)
Supplement: Supplementary file 1 [file DataSheet_1.pdf]

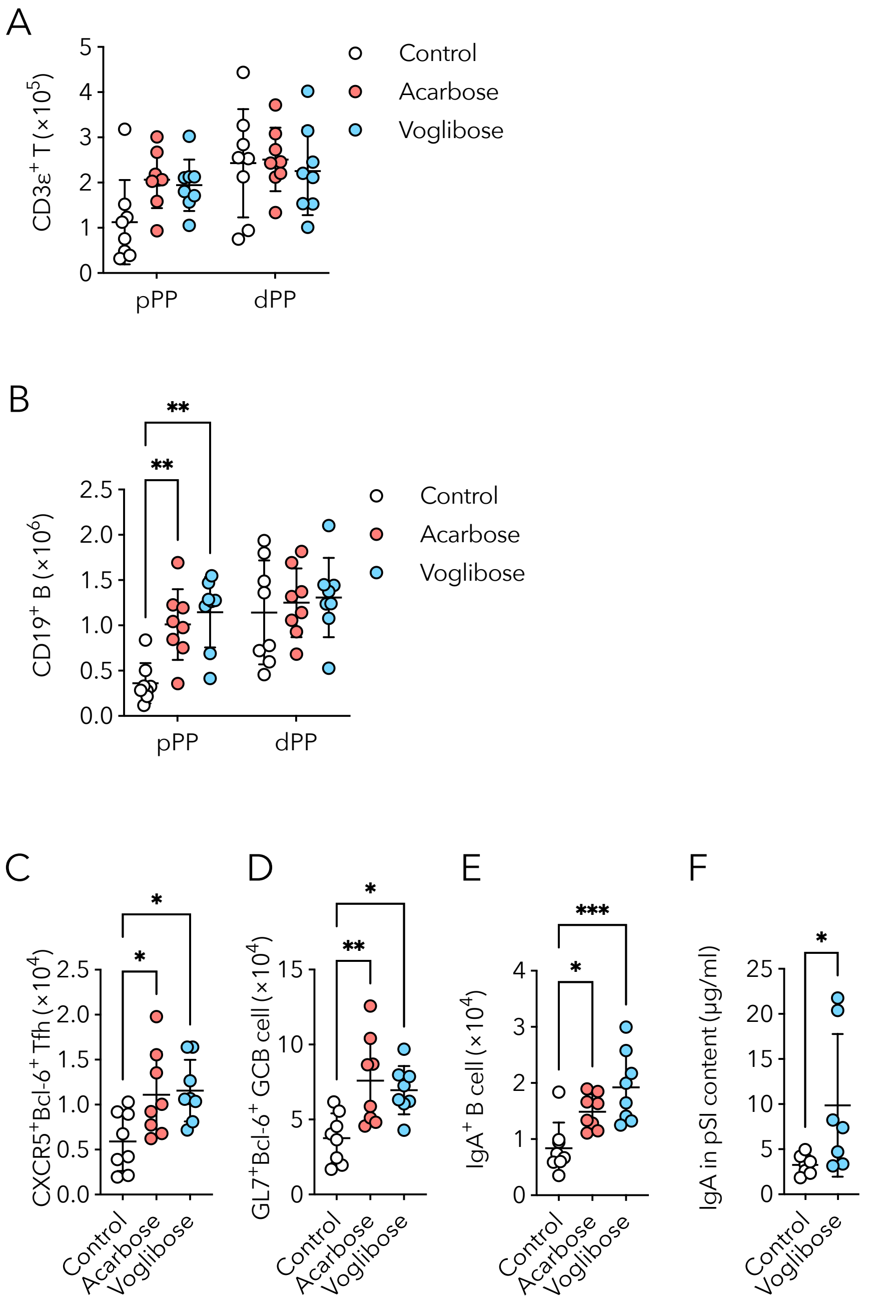

Supplementary Figure 1 | α-GI acarbose and voglibose increase Tfh, GCB, and IgA-producing B cells. (**A, B**) Number of CD3ε⁺ T cells (**A**) and CD19⁺ B cells (**B**) in PPs of the proximal and distal part of the small intestine (pPP, dPP respectively) in the control mice and mice treated with 0.125% acarbose, and 0.0005% voglibose via drinking water (n = 8, means ± s.d.). (**C–E**) Number of CXCR5⁺Bcl-6⁺ Tfh cells (**C**), Bcl-6⁺GL-7⁺ GCB cells (**D**), and IgA⁺ B cells (**E**) in PPs of entire small intestine in the control mice and mice treated with 0.125% acarbose, and 0.0005% voglibose via drinking water (n = 8, means ± s.d.). (**G**) The concentration of IgA in proximal small intestinal content (n = 7, means ± s.d.). Mice were administered 0.00025% voglibose (n = 7, means ± s.d.) via drinking water for 4 weeks starting from 3 weeks of age. *P < 0.05, **P < 0.01, ***P < 0.001 (A–E, two-way or on–way ANOVA followed by Dunnet's post-hoc test; F, Welch's t-test)

A

B

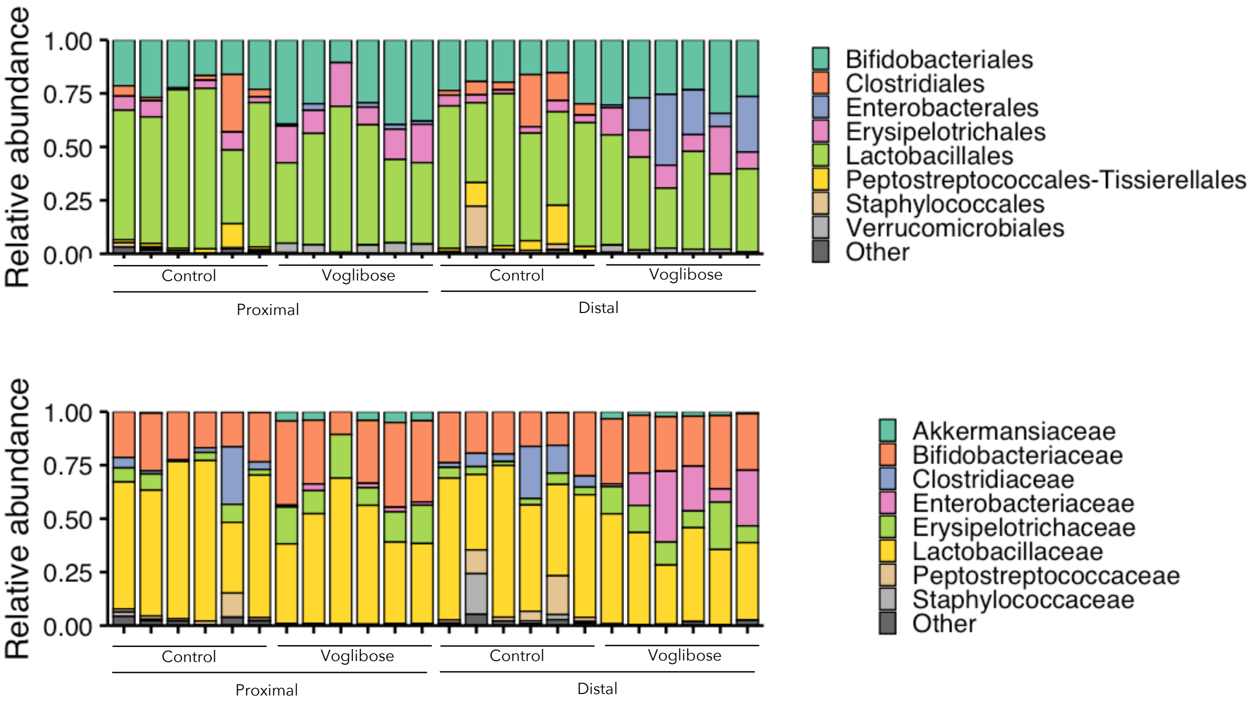

Supplementary Figure 2 | Voglibose alters the composition of commensal microbiota in the small intestine. (**A, B**) The composition of microbiota at the order (**A**) and family (**B**) levels in the small intestinal content from the proximal and distal part of the small intestine in the control mice and mice treated with 0.00025% voglibose.

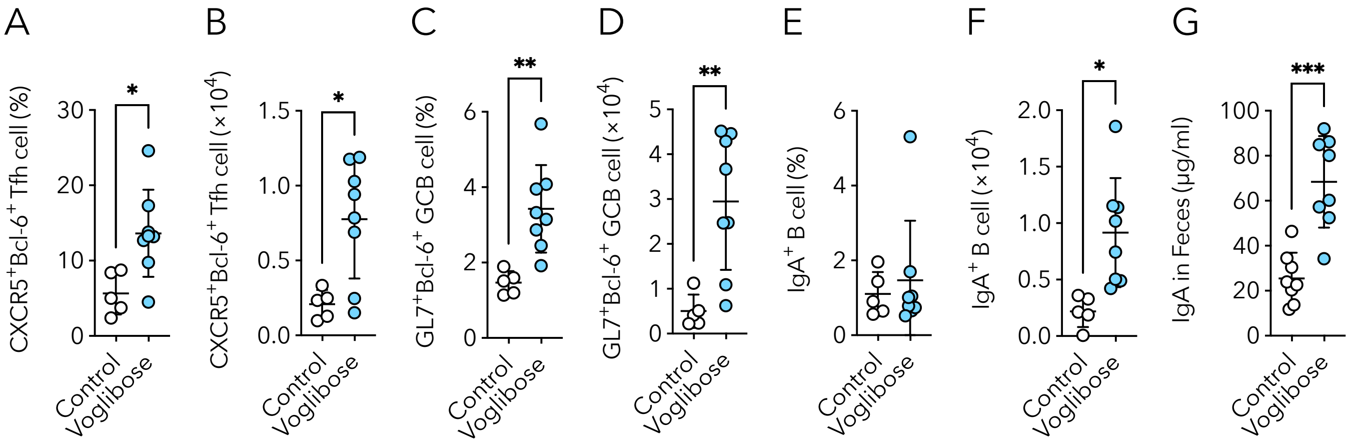

Supplementary Figure 3 | α-GI voglibose increases Tfh, GCB, and IgA-producing B cells in female mice. (**A, B**) The frequency (**A**) and the total number (**B**) of $CXCR5^+Bcl\text{-}6^+$ Tfh cells in PPs of the proximal part of the small intestine (n = 6, 8; means ± s.d.). (**C, D**) The frequency (**C**) and the total number (**D**) of $Bcl\text{-}6^+GL\text{-}7^+$ GCB cells in PPs of the proximal part of the small intestine in the control mice (n = 6, 8; means ± s.d.). (**E, F**) The frequency (**E**) and the total number (**F**) of IgA^+ B cells in PPs of the proximal part of the small intestine (n = 6, 8; means ± s.d.). (**G**) The concentration of IgA in fecal samples (n = 8; means ± s.d.). Female mice were administered 0.00025% voglibose via drinking water for 4 weeks starting from 3 weeks of age. Results show one representative experiment of at least two experiments. *$P < 0.05$, ** $P < 0.01$, *** $P < 0.001$ (A–G, Student's t-test or Welch's t-test)

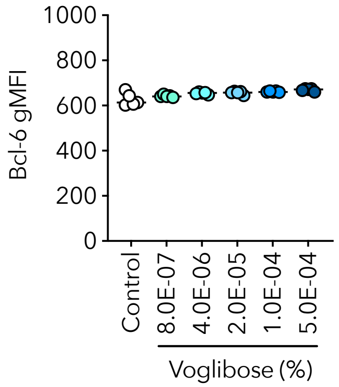

Supplementary Figure 4 | Voglibose treatment slightly upregulated the expression of Bcl-6. Naïve $CD4^+$ T cells were cultured *in vitro* for 5 days under Tfh-cell-skewing conditions in the absence or presence of voglibose treatment. Bcl-6 expression was analyzed by flow cytometry. (one-way ANOVA followed by Dunnet's post-hoc test; G, Student's t-test).

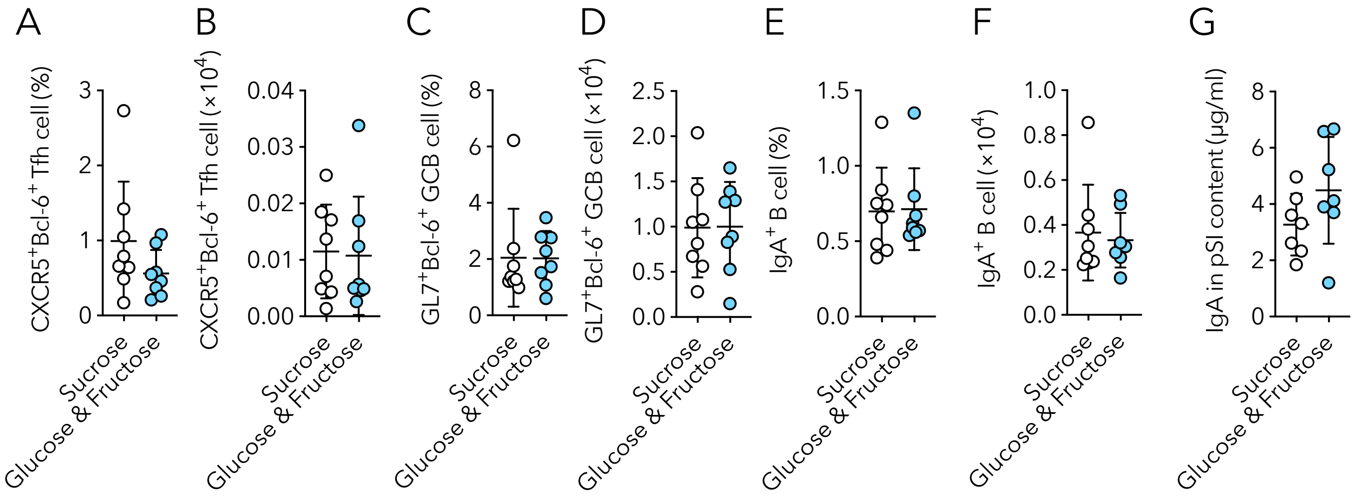

Supplementary Figure 5 | The replacement of sucrose with D(+)-glucose and D(-)- fructose shows no effect on Tfh, GCB, and IgA-producing B cells. (**A, B**) The frequency (**A**) and the total number (**B**) of CXCR5$^+$Bcl-6$^+$ Tfh cells in PPs of the proximal part of the small intestine (*n* = 8; means ± s.d.). (**C, D**) The frequency (**C**) and the total number (**D**) of Bcl-6$^+$GL-7$^+$ GCB cells in PPs of the proximal part of the small intestine in the control mice (*n* = 8; means ± s.d.). (**E, F**) The frequency (**E**) and the total number (**F**) of IgA$^+$ B cells in PPs of the proximal part of the small intestine (*n* = 8; means ± s.d.). (G) The concentration of IgA in the proximal small intestine content (*n* = 8; means ± s.d.). Mice were fed either a sucrose-containing basal AIN-93G diet or a sucrose-free AIN-93G diet containing D(+)-glucose and D(-)-fructose (5% w/w each) for 4 weeks starting from 3 weeks of age. (A–G, Student's t-test or Welch's t-test)

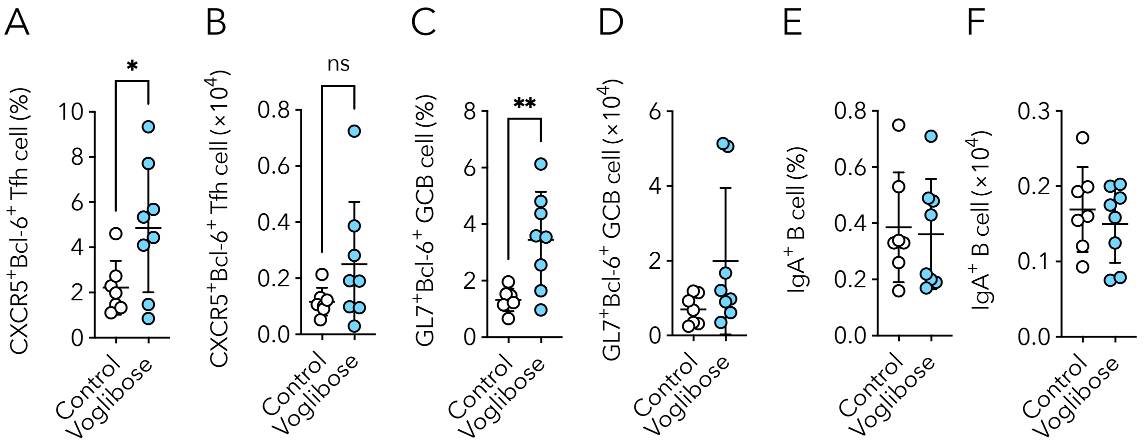

Supplementary Figure 6 | α-GI voglibose increases Tfh cells, GCB cells, and total IgA levels in proximal small intestinal content during Salmonella infection. (**A, B**) The frequency (**A**) and the total number (**B**) of CXCR5$^+$Bcl-6$^+$ Tfh cells in PPs of the proximal part of the small intestine (n = 7, 8; means ± s.d.). (**C, D**) The frequency (**C**) and the total number (**D**) of Bcl-6$^+$GL-7$^+$ GCB cells in PPs of the proximal part of the small intestine in the control mice (n = 7, 8; means ± s.d.). (**E, F**) The frequency (**E**) and the total number (**F**) of IgA$^+$ B cells in PPs of the proximal part of the small intestine (n = 7, 8; means ± s.d.). Mice were administered 0.00025% voglibose via drinking water for 2 weeks initiating from 3 weeks of age, followed by infection with Salmonella-ToxC (ΔaroA, ΔaroD). Subsequently, administration of 0.00025% voglibose was continued for another 2 weeks. *P < 0.05, **P < 0.01 (A–G, Student's t-test or Welch's t-test)

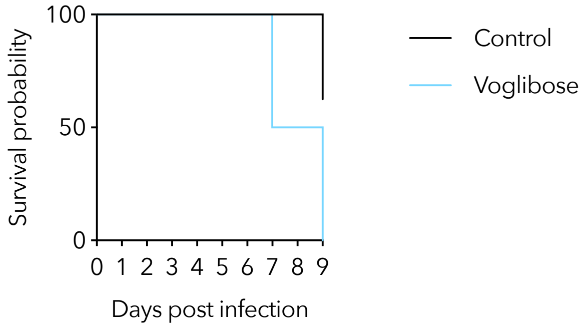

Supplementary Figure 7 | Survival probability after *Salmonella* Typhimurium infection was comparable between control and voglibose-treated mice. Mice were administered 0.00025% voglibose in drinking water for 2 weeks from 3 weeks of age and were orally infected with approximately 1×10^6 colony-forming unit (CFU) of *Salmonella enterica* subsp. *enterica* serovar Typhimurium χ3181. (Log-rank test and Gehan-Breslow-Wilcoxon test)

Supplementary Table 1 | Composition of AIN-93G (basal) diet and modified AIN-93G diet

%	AIN-93G (basal)	AIN-93G (glucose)	AIN-93G (glucose & fructose)
casein	20	20	20
L-cystine	0.3	0.3	0.3
cornstarch	34.7486	34.7486	34.7486
α-cornstarch	13.2	13.2	13.2
sucrose	10	0	0
D(+)-glucose	0	10	5
D(-)-fructose	0	0	5
soy oil	7	7	7
cellose powder	5	5	5
AIN-93G mineral mix	3.5	3.5	3.5
AIN-93G vitamin mix	6	6	6
Choline bitartrate	0.25	0.25	0.25
tertiary butyl hydroquinc	0.0014	0.0014	0.0014
total	100	100	100

Supplementary Table 1 | Spearman's rank correlation between fecal IgA concentration and commensal bacterial families in the small intestinal content of mice treated with voglibose.

Proximal small intestinal content		
Variable	ρ	p
Atopobiaceae	0.393	0.441
Streptococcaceae	0.030	0.954
Enterobacteriaceae	0.029	0.957
Lachnospiraceae	-0.034	0.949
Lactobacillaceae	-0.086	0.872
Enterococcaceae	-0.087	0.870
Akkermansiaceae	-0.143	0.787
Bifidobacteriaceae	-0.257	0.623
Staphylococcaceae	-0.393	0.441
Erysipelotrichaceae	-0.543	0.266
Distal small intestinal content		
Variable	ρ	p
Lactobacillaceae	0.886	0.019
Streptococcaceae	0.488	0.326
Atopobiaceae	0.393	0.441
Akkermansiaceae	0.257	0.623
Enterococcaceae	0.152	0.774
Enterobacteriaceae	-0.200	0.704
Erysipelotrichaceae	-0.406	0.425
Bifidobacteriaceae	-0.429	0.397
Lachnospiraceae	-0.845	0.034

Supplementary Materials and methods

In vitro Tfh cell differentiation and flow cytometry

CD4$^+$ cells were enriched from the spleen by negative selection using MojoSort Mouse CD4$^+$ T Cell Isolation Kit (BioLegend) according to the manufacturer's instructions. Briefly, single-cell suspension was incubated with a mixture of biotin-conjugated mAb cocktail in staining buffer (PBS containing 2% FCS with 2 mM EDTA) for 15 minutes at 4°C. Streptavidin Nanobeads were then added and incubated for 15 minutes at 4°C. The enriched CD4$^+$ fraction was stained with 7-AAD (BioLegend) and mAbs including Brilliant Violet (BV)605-conjugated anti-mouse CD4 (RM4-5; BioLegend), BV510-conjugated anti-mouse (30-F11; BioLegend), BV786-conjugated anti-mouse CD62L (MEL-14; BD Biosciences), BB515-conjugated anti-mouse CD25 (PC61; BD Biosciences), and APC-conjugated anti-mouse CD44 (IM7; BD Biosciences). Stained cells were subjected to cell sorting using a FACSAria III cell sorter to isolate live CD45$^+$CD4$^+$CD44^{lo}CD62^{hi}CD25$^-$ naïve T cells.

Naïve sort-purified CD4$^+$ T cells (5×10^5 cells/ml) were stimulated with immobilized anti-mouse TCRβ mAb (H57-597; BioLegend, 5 μg/ml) on a high-binding 96-well plate (Corning, Corning, NY, USA) and soluble anti-mouse CD28 mAb (37.51; BioLegend, 2 μg/ml) in complete RPMI 1640 media (Thermo Fisher Scientific) containing 10% v/v fetal calf serum (FCS; MP Biomedicals, Santa Ana, CA, USA) supplemented with 0.5 ng/ml recombinant human TGF-β1, 50 ng/ml recombinant mouse (rm) IL-6, 10 μg/ml anti-mouse IL-4 (11B11, BioLegend), and 10 μg/ml anti-mouse IFN-γ (R4-6A2, BioLegend) for two days. The stimulated T cells were expanded in complete media supplemented with 50 ng/ml rmIL-6, 50 ng/ml rmIL-21, 10 μg/ml anti-mouse ICOS (7E.17G9, BioLegend), 10 μg/ml anti-mouse IL-4 (11B11, BioLegend), and 10 μg/ml anti-mouse IFN-γ (R4-6A2, BioLegend) for another three days.

Cultured T cells were pre-incubated with a monoclonal antibody (mAb) against CD16/32 (S17011E; BioLegend) in staining buffer (PBS containing 2% FCS and 0.1% NaN_3) before surface antigen staining. Tfh-cell staining was performed with mAbs including 605-conjugated anti-mouse CD4 (RM4-5; BioLegend), BV650-conjugated anti-mouse TCRβ-chain (H57-597; BD Biosciences), and BV510 (BV510)-conjugated anti-mouse CD45 (30-F11; BioLegend), followed by dead cell staining with Fixable Viability Stain 780 (FVS780; BD Biosciences). The cells were then fixed, permeabilized, and stained with mAbs including PE-conjugated anti-Bcl-6 (K112-91; BD Biosciences) using a transcription factor buffer set (BD Biosciences). Flow cytometry was performed using a FACSCelesta flow cytometer with DIVA v9.0 (BD Biosciences), and data were analyzed using FlowJo version 10.9 (BD Biosciences).

Oral infection of wild-type *S. Typhimurium*

Mice were administered 0.00025% voglibose in drinking water for 2 weeks from 3 weeks of age and were orally infected with approximately 1×10^6 colony-forming unit (CFU) of *Salmonella enterica* subsp. *enterica* serovar Typhimurium χ3181.
